# The impact of smoke-free legislation on the illegal cigarette trade in China: A longitudinal study

**DOI:** 10.18332/tid/219212

**Published:** 2026-07-26

**Authors:** Mengting Xu, Zili Zhang, Xiao Hu, Rong Zheng

**Affiliations:** 1 School of Public Finance and Taxation, Zhejiang University of Finance and Economics, Zhejiang, China; 2 Zhejiang Province ‘Eight-Eight Strategy’ Innovative Development Think Tank Alliance, Zhejiang Province ‘Eight-Eight Strategy’ Research Institute, Hangzhou, China; 3 School of Public Finance and Taxation, Nanjing University of Finance and Economics, Nanjing, China; 4 School of International Trade and Economics, University of International Business and Economics, Beijing, China; 5 WHO Collaborating Centre on Health Tax and Fiscal Policy, Beijing, China

**Keywords:** cigarette, smoke-free legislation, illegal cigarette trade

## Abstract

**INTRODUCTION:**

Although numerous studies have examined the relationship between smoke-free legislation and cigarette consumption, research focusing specifically on its impact on the illicit cigarette trade remains scarce. This study explores the effects of subnational smoke-free legislation on the illicit cigarette trade in China.

**METHODS:**

Using a balanced panel dataset of nearly 300 Chinese cities from 2011 to 2016, we first extract information on illicit tobacco seizures from the administrative records of the State Tobacco Monopoly Administration (STMA). We then employ a fixed-effects model to analyze the impact of smoke-free legislation on local illicit cigarette trade. To address endogeneity, we conduct robustness tests using propensity score matching and instrumental variables methods.

**RESULTS:**

We find that smoke-free legislation has a significant negative impact on the illicit cigarette trade. Specifically, regression results indicate that the legislation reduces local illicit trade by an average of 26.65% (β= -0.2665; 95% CI: -0.50- -0.02; p=0.026). Heterogeneity analysis reveals that this effect is more pronounced in coastal and border regions compared to inland areas.

**CONCLUSIONS:**

These findings suggest that non-price tobacco control measures can effectively suppress the illicit market through a supply-side deterrence mechanism. However, further longitudinal studies are required across diverse institutional contexts to obtain sufficient evidence to inform global anti-smuggling strategies.

## INTRODUCTION

Tobacco consumption remains a leading cause of preventable death worldwide^1^, prompting governments to implement stringent control measures, including tax hikes and smoke-free legislation^2^. While the public health benefits of these policies are well-documented, a persistent debate in health economics and policy circles concerns their potential unintended consequences – specifically, the proliferation of the illicit cigarette trade^3^. Economic theory suggests that stricter regulations often raise compliance costs, potentially driving consumers toward the black market^4^. Consequently, critics argue that aggressive regulation might inadvertently fuel smuggling activities, thereby undermining both public health objectives and fiscal revenues^3,5^. Therefore, understanding whether smoke-free legislation acts as a catalyst for – or a deterrent to – illicit trade is a critical empirical question with significant policy implications.

The efficacy of such regulatory measures depends heavily on the institutional and socio-economic environment. While extensive literature identifies factors such as economic adversity and legal enforcement strength as primary drivers of economic crimes^6,7^, research specifically linking non-price measures like smoke-free legislation to illicit trade remains scarce. Existing studies have focused predominantly on price-based interventions^8-10^. Recent evidence from China, for instance, indicates that tobacco tax hikes can inadvertently stimulate smuggling by increasing the expected profit margins for offenders^11^. In contrast, we posit that smoke-free legislation operates through a distinct mechanism: rather than altering profit incentives, it targets the cost side by raising transaction costs and the risks of detection. While the existing smoke-free legislation literature has focused predominantly on demand-side responses^12-15^, recent evidence from China has begun to highlight the policy’s broader macro-economic outcomes beyond individual health behaviors^16^. However, the policy’s potential to reshape the supply-side dynamics of the illicit market remains largely unexplored.

This study bridges this gap by providing causal evidence from China, the world’s largest tobacco market^17^. China offers an ideal setting for this analysis due to its massive tobacco market and significant regional variation in policy enforcement^18-21^. Historically, the State Tobacco Monopoly Administration (STMA) provided detailed administrative records regarding illicit tobacco seizures, which offer a comprehensive view of the market’s supply-side dynamics^22^. By utilizing these authoritative records from a period of high data transparency, the primary aim of this study is to investigate the causal impact of smoke-free legislation on the illicit cigarette trade. Specifically, we examine whether these non-price regulatory measures effectively reduce illicit trade activities. Furthermore, this study aims to explore the potential underlying mechanisms – such as transaction costs and enforcement capacity – by analyzing variations across diverse geographical and institutional contexts. By addressing these objectives, this research seeks to clarify the role of smoke-free policies within the broader framework of tobacco control and provide evidence-based insights for global public health strategies.

## METHODS

### Data

This article presents a longitudinal study based on panel data from 283 prefecture-level cities in China covering the period from 2011 to 2016. These 283 prefecture-level cities account for 96.59% of all prefecture-level cities in China, making the sample representative. Data on cigarette sales volumes in various cities are manually extracted from the China Tobacco Yearbook and annual work reports published by the State Tobacco Monopoly Administration (STMA). Following the research by Zhang and Zhang^11^, we also utilized data from 2011 to 2016. The second database is the China City Statistical Yearbook. This database provides the basic socio-economic information of a city^23^, such as total population, per capita gross domestic product (GDP), industrial structure, etc. We matched the two datasets year by year to form the panel data required for empirical analysis.

### Variables

The dependent variable in this study is the number of illicit cigarette trade cases, measured by the number of tobacco-related violations detected by local tobacco companies and local governments. Since there is no distinction between fake cigarettes and smuggled real cigarettes in cases of tobacco-related violations, the number of cases of cigarette crimes at the prefecture and municipal levels used in this study are the sum of cases of fake cigarettes and smuggled real cigarettes.

The main predictor variable of interest is *Policy*_it_, whose coefficient captures the effect of the smokefree legislation policy on cigarette crime. *Policy*_it_ is a time dummy variable that takes a value of 1 when city *i* implements the smoke-free legislation in year *t*, and 0 otherwise.

We control for other important variables that could potentially influence the illicit cigarette trade. Following the methodology of Zhang and Zhang^9^, we controlled for local cigarette demand, cigarette control intensity, economic level, population size, and industrial structure. Specifically, cigarette demand represents local cigarette demand, which we measure using cigarette sales volume. Cigarette employees is the number of employees at local tobacco bureaus in each city, which can serve as a measure of regulatory intensity against illicit cigarette trade. Population represents the population size of each city, measured by the year-end total population from the city’s statistical yearbook. Per capita GDP indicates the local economic level, measured by local per capita GDP. Industrial structure refers to the local industrial structure, measured by the proportion of the tertiary industry value-added in local GDP.

To address potential endogeneity issues, we employ the instrumental variables (IV) approach. The instrumental variable in this study is Civilized City, which refers to whether a city received the national title of Civilized City prior to 2010.

### Statistical analysis

We employed three methodologies to analyze the impact of smoke-free legislation on the illicit trade of cigarettes. All analyses were conducted using Stata 19.

First, we employed a two-way fixed effects model for analysis. This econometric tool, commonly used in panel data analysis, simultaneously controls for individual effects and time effects, thereby enabling more accurate estimation of regression coefficients. To investigate the influence of smoke-free legislation on illicit cigarette trade, we utilize the following regression equation:

*Y_ij_ = a_0_ + β_0_Policy_it_ + β_1_X_it_ +τ_t_+ u_i_ + ε_it_*

Our dependent variable *Y*_it_ measures the number of illicit cigarette trade cases in city *i*, year *t*. *Policy*_it_ is a dummy variable indicating whether city *i* implemented the smoke-free legislation policy in year *t*. The coefficient *β_0_* is our focus, representing the impact of smoke-free legislation policy on the illegal cigarette trade. *X*_it_ represents a set of control variables, including cigarette demand, cigarette employees, per capita GDP, population, industrial structure etc., and *u*_i_ and *τ*_t_ represent the city fixed effect and time fixed effect, respectively. Additionally, the constant term *a*_0_ typically represents the average baseline level of the dependent variable when no predictor variables or fixed effects are considered. The error term *ε*_it_ accounts for the residual variation that cannot be explained by the predictor variables, city fixed effects, or time fixed effects.

Second, propensity score matching (PSM) was conducted to control for confounding factors, including cigarette demand, cigarette employees, per capita GDP, population, and industrial structure. We performed 1:3 propensity score matching without replacement using a caliper of 0.1 SD of the propensity score logit. Based on propensity score matching values, city samples exhibiting characteristics similar to those of smoke-free legislation cities were matched from the non-smoke-free legislation city sample. Subsequently, we conducted regression analysis using the successfully matched samples.

Third, we adopted the instrumental variables (IV) approach. Since the potential problems of omitted variables and endogeneity of mutual causality in a two-way fixed effects model (FE model), an instrumental variable approach is adopted. An effective instrumental variable must be highly correlated with the endogenous explanatory variable and independent of the error term. We employed the designation of National Civilized City prior to 2010 as an instrumental variable for assessing cities’ implementation of smoke-free legislation. China’s National Civilized City selection began in 2005 as an honorary title conferred by the Chinese government on cities demonstrating outstanding performance in intellectual development, aiming to elevate overall urban civility and citizen quality.^[24]^ Being selected as a National Civilized City signifies a higher level of civic culture, making it more likely for the city to implement smoke-free legislation, thus satisfying the requirements for instrumental variable relevance. Furthermore, the designation as a National Civilized City prior to 2010 is exogenous to the illicit cigarette trade. Overall, our instrumental variable satisfies both relevance and exogeneity.

## RESULTS

### Descriptive statistics

Table 1 presents the summary statistics of key variables. Among the 283 cities in the sample, 94.35% (n=267) of prefecture-level cities have enacted smoke-free legislation, while 5.65% (n=16) have not. We have presented the mean and standard deviation for each key variable across the total sample (n=1698), the smoke-free legislation cities sample (n=96), and the non-smoke-free legislation cities sample (n=1602) for the years 2011–2016 in three separate columns. The average population was 4.2928 million, with a per capita GDP of 47100 RMB (1000 Chinese Renminbi about US$150). The tertiary industry’s value-added contribution averaged 38% of GDP. National Civilized Cities accounted for 11% of the total sample. Furthermore, we conducted t-tests on the variables between the smoke-free legislation cities sample and the non-smoke-free legislation cities sample. It can be seen that significant differences exist in the variable values between the two groups.

**Table 1:** Descriptive statistics of major variables, 2011–2016 (N=1698)

*Variables*	*All* *(N=1698)* *Mean (SD)*	*Smoke-free legislation cities* *(N=96)* *Mean (SD)*	*Non-smoke-free legislation cities* *(N=1602)* *Mean (SD)*
**Dependent**			
Illicit cigarette trade cases	1381.01 (1567.71)	2553.91*** (2200.3)	1310.77 (1493.49)
**Control**			
Cigarette demand	76.42 (53.03)	138.78*** (67.10)	72.69 (49.67)
Cigarette employees	823.77 (616.51)	1118.63*** (554.64)	806.10 (615.71)
Per capita GDP	4.71 (3.06)	7.94*** (2.83)	4.52 (2.96)
Population	429.28 (260.39)	609.17*** (241.14)	418.49 (257.61)
Industrial structure	0.38 (0.09)	0.48*** (0.06)	0.38 (0.09)
**Instrumental**			
Civilized City	0.11 (0.31)	0.44*** (0.50)	0.09 (0.29)

Units of cigarette demand, per capita GDP, and population are 100 million cigarettes, 10 thousand RMB, and 10 thousand people, respectively. Illicit cigarette trade cases and cigarette employees represent the actual number of illegal cigarette trade cases and the number of employees of the State Tobacco Monopoly Administration, respectively. RMB: 1000 Chinese Renminbi about US$150. Civilized City signifies a higher level of civic culture.

*** p<0.01, ** p<0.05, * p<0.1.

### Impact assessment based on three approaches

Table 2 reports the regression results on the impact of smoke-free legislation on the illicit cigarette trade, analyzed using three different methodologies. Column 2 of Table 2 presents the regression results based on the fixed-effects model (FE). To eliminate the effect of differences in measurement units across variables, we take the logarithm of illicit cigarette trade cases, cigarette demand, cigarette employees, per capita GDP, and population in the regression analysis. The two-way fixed effects model shows that smoke-free legislation significantly decreases the number of illicit cigarette trade cases (β= -0.2665; 95% CI: -0.50- -0.02; p=0.026).

**Table 2:** The effects of smoke-free legislation on illicit cigarette trade, 2011-2016

*Variables*	*Model 1 (FE)* *β (95% CI)*	*Model 2 (PSM)* *β (95% CI)*	*Model 3 (IV)* *β (95% CI)*
Policy	-0.2665** (-0.50- -0.02)	-0.6514*** (-1.02 – -0.29)	-1.8313** (-3.89–0.23)
p	0.026	0.001	0.081
Log cigarette demand	0.6179*** (0.33–0.89)	0.3620 (-0.21–0.94)	0.9283*** (0.77–1.08)
p	<0.001	0.215	<0.001
Log cigarette employees	0.3001*** (0.15–0.44)	0.1481 (-0.16–0.46)	0.0450 (-0.05–0.14)
p	<0.001	0.347	0.376
Log per capita GDP	-0.2415** (-0.46–0.02)	-0.0119 (-0.52–0.49)	0.1549* (-0.02–0.33)
p	0.032	0.963	0.084
Log population	0.3845 (-0.29–1.07)	0.4582 (-2.09–3.00)	-0.0009 (0.000–0.001)
p	0.270	0.724	<0.001
Industrial structure	-1.3719** (-2.52 – -0.22)	1.4237 (-1.67–4.51)	-0.3942 (-1.32–0.53)
p	0.019	0.365	0.402
Year and city fixed effects	Yes	Yes	Yes
Observations	1698	390	1692
R^2^	0.0434	0.0537	NA
First-stage F-statistic	NA	NA	20.68

FE: two-way fixed effects model. PSM: propensity score matching method. IV: instrumental variable method. All regressions include year and city fixed effects. NA: not applicable.

*** p<0.01,

** p<0.05,

* p<0.1.

**Figure 1:**
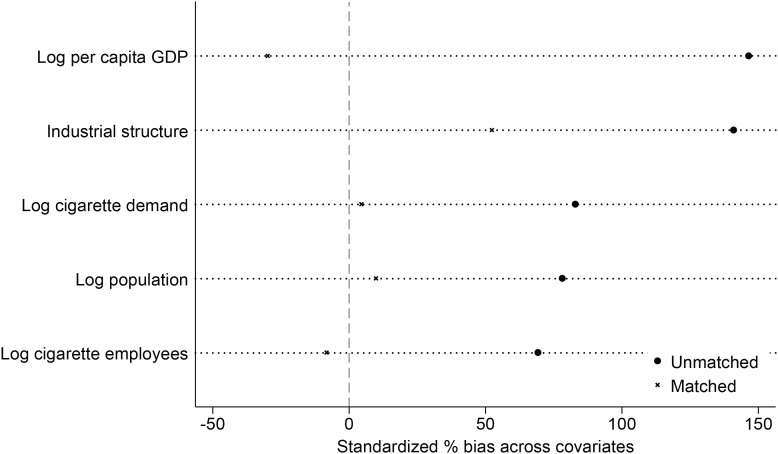
Balance of covariates before and after propensity score matching

The regression results of the fixed-effects model after PSM are shown in column 3 of Table 2. Before PSM, 1698 observations were included; after PSM, 390 matched observations remained. Post-PSM analysis eliminated differences in baseline covariates between smoke-free cities and non-smoke-free cities (Figure 1). Statistical analysis of the matched dataset revealed that smoke-free legislation has a significant negative impact on the number of illegal cigarette trade cases (β = -0.6514; 95% CI: -1.02 – -0.29; p=0.001).

Column 4 in Table 2 shows the regression results of the IV method. The Cragg-Donald Wald F-statistic of the first stage regression is >10, indicating that our IV passes the weak IV identification test (F-statistic=20.68). The regression results indicate that smoke-free legislation exerts a significant negative impact on the illicit trade of cigarettes (β= -1.8313; 95% CI: -3.89 – -0.23; p=0.081). The results are consistent with the estimates from the FE method and PSM method, indicating that our conclusions are robust.

Prior to PSM, significant differences were observed in the absolute standardized means of confounding factors. After PSM, these differences were effectively eliminated, ensuring balanced baseline characteristics between the smoke-free cities sample and the non-smoke-free cities sample, matched on: Log cigarette demand, Log cigarette employees, Log per capita GDP, Log population, and industrial structure.

### Heterogeneity analysis

Table 3 presents the results of the grouped regression analysis based on the fixed-effects model. We divided the sample into two subgroups, border and coastal cities (n=63) and inland cities (n=219), and performed separate regressions. Heterogeneity analysis indicates that the negative impact of smoke-free legislation on illicit cigarette trade in coastal and border regions is statistically significant at 1% level (β= -1.0792; 95% CI: -1.72 – -0.43; p=0.001), while its effect on illicit cigarette trade in inland areas is insignificant (β= -0.1688; 95% CI: -0.42 – -0.08; p=0.192).

**Table 3:** The heterogeneous effects of smoke-free legislation on illicit cigarette trade, 2011–2016

*Variables*	*Coastal and border cities* *β (95% CI)*	*Inland cities* *β (95% CI)*
Policy	-1.0792*** (-1.72 – -0.43)	-0.1688 (-0.42–0.08)
p	0.001	0.192
Log cigarette demand	-0.1146 (-0.76–0.53)	0.6521*** (0.34–0.96)
p	0.727	<0.001
Log cigarette employees	0.4846*** (0.20–0.77)	0.2484*** (0.08–0.42)
p	0.001	0.004
Log per capita GDP	-0.1795 (-0.59–0.24)	-0.2514* (-0.51 – -0.01)
p	0.400	0.057
Log population	5.7797*** (3.41–8.14)	0.0174 (-0.71–0.74)
p	<0.001	0.962
Industrial structure	-1.9432 (-4.38–0.49)	-0.9626 (-2.28–0.35)
p	0.118	0.151
Year and city fixed effects	Yes	Yes
Observations	378	1314
R^2^	0.2113	0.0386

All regressions include year and city fixed effects.

*** p<0.01, ** p<0.05,

* p<0.1.

## DISCUSSION

Building on the empirical results, this study provides empirical evidence that non-price tobacco control measures, specifically smoke-free legislation, effectively suppress the illicit cigarette trade in China. Our primary finding, a significant decline in local cigarette-related violation cases, challenges the conventional critique that strict regulations inevitably fuel the black market^10,25^. This divergence is best understood through the rational choice framework of crime economics, where offenders weigh expected benefits against legal risks and transaction costs^26-28^. While traditional price-based measures, such as the 2015 tobacco excise tax hike, may inadvertently stimulate illicit trade by widening profit margins for smugglers in certain institutional settings^11^, our results suggest that smoke-free legislation targets the opposing side of the criminal calculus by increasing the risks and costs of distribution.

This suppressive effect operates primarily through a supply-side deterrence mechanism rather than a mere contraction in tobacco demand. By explicitly controlling for legal cigarette sales in our baseline model, we isolate the policy’s impact from shifts in aggregate consumption. This indicates that the observed decline in illicit trade is not simply a byproduct of a shrinking consumer base, but rather reflects a disruption of the underground supply chain. The implementation of smoke-free laws typically leads to intensified surveillance and monitoring of public spaces, such as restaurants, transportation hubs, and office buildings, reflecting a broader shift toward comprehensive urban health governance^20^. This improved urban governance creates a regulatory spillover, making it increasingly difficult for vendors to distribute or sell illegal products in these now-monitored environments without detection. This aligns with the evidence suggesting that non-price regulations can serve as efficient tools for market purification in tobacco-heavy economies^2,10^.

To validate this deterrence interpretation, it is crucial to address the potential for resource diversion within the Chinese administrative system – a point central to the debate over regulatory efficiency. A common concern in policy evaluation is whether the decrease in detected cases reflects a genuine reduction in crime or a weakening of enforcement due to resource reallocation. However, our study highlights a unique dual-track institutional arrangement in China: the enforcement of smoke-free legislation is primarily managed by health departments, whereas anti-smuggling operations remain the sole responsibility of the STMA^19,^ ^29^. This functional separation minimizes the likelihood of direct personnel displacement. Furthermore, by quantitatively controlling for the number of STMA employees at the city level, we confirm that the reduction in violation cases persists even when holding actual regulatory capacity constant, thereby dismissing the resource diversion hypothesis.

The robustness of this deterrence mechanism is further substantiated by the geographical heterogeneity observed in our analysis. We find that the suppressive impact of smoke-free legislation is most pronounced in coastal and border regions. Under China’s tobacco monopoly system, these areas have historically served as the primary conduits for both counterfeit and smuggled cigarettes due to their logistical advantages^22^. The fact that smoke-free legislation achieves its most substantial impact in these high-intensity smuggling zones suggests that the legislation effectively strengthens regulatory leverage precisely where the underground market is most entrenched. This aligns with recent evidence suggesting that these hubs are particularly sensitive to shifts in the risk–reward ratio of illicit tobacco flows^11^.

Ultimately, the empirical evidence suggests that our estimated reduction represents a conservative lower bound of the actual policy impact. In the context of improved urban governance, a more intense monitoring environment should theoretically lead to a mechanical increase in seizures if the volume of illegal trade remained constant^10^. The fact that we observe a significant and robust decrease – even while accounting for enforcement resources and tobacco demand – implies that the true reduction in the volume of illicit trade is substantial enough to override this detection-bias. Consequently, in the specialized context of China’s tobacco monopoly, smoke-free legislation serves as a highly efficient regulatory instrument that complements traditional enforcement by increasing the risk premium for illegal operations.

### Limitations

Our research has limitations. Due to the discontinuation of specific public data disclosure by the STMA, as documented in the China Tobacco Yearbook series, our analysis is constrained to the period ending in 2016. Future research, should more recent administrative data become available, could examine the long-term persistence of these effects or explore the potential synergies between smokefree legislation and more recent digital tracking technologies in tobacco enforcement. Additionally, our findings are primarily based on the Chinese context, and their applicability to other countries requires further investigation.

## CONCLUSIONS

Smoke-free legislation significantly reduces the frequency of tobacco-related violation cases by establishing a supply-side deterrence mechanism that increases the costs and risks for illegal traders. This observed reduction represents a conservative lower bound of the actual policy impact, as it overrides the mechanical increase in detections typically associated with improved urban governance. While these findings suggest that non-price regulations can effectively complement anti-smuggling efforts, further longitudinal studies and cross-regional comparisons are required to build a more comprehensive evidence base. Such research is essential to fully understand the long-term persistence of these effects and to provide sufficient evidence necessary to inform the integration of smoke-free legislation into broader tobacco control and anti-smuggling strategies.

## Data Availability

The data supporting this research are available from the authors on reasonable request.
